# Retrieval of Polyphenols Using Aqueous Two-Phase Systems Based on Ethyl Lactate and Organic Salts

**DOI:** 10.3390/molecules30071532

**Published:** 2025-03-30

**Authors:** Gonçalo Perestrelo, Pedro Velho, Eugénia A. Macedo

**Affiliations:** 1 Laboratory of Separation and Reaction Engineering—Laboratory of Catalysis and Materials (LSRE-LCM), Faculty of Engineering, University of Porto, Rua Dr. Roberto Frias, 4200-465 Porto, Portugal; gperestrelo@fe.up.pt; 2 Associate Laboratory in Chemical Engineering (ALiCE), Faculty of Engineering, University of Porto, Rua Dr. Roberto Frias, 4200-465 Porto, Portugal

**Keywords:** waste valorisation, bioactive substances, liquid–liquid extraction, ATPS, ethyl lactate

## Abstract

Food waste remains a critical global concern, with approximately one third of all food produced being ultimately discarded. Therefore, it is urgent to develop new techniques for the effective repurpose of waste. Aqueous two-phase systems (ATPSs) stand out as a simple and biocompatible liquid–liquid extraction technique for the recovery of bioactive substances from food waste. In ATPSs, the target species partition between two liquid phases, according to affinity, which facilitates its extraction. This work aimed at extracting three polyphenols—chlorogenic acid (CA), ferulic acid (FA), and resveratrol (RV)—through the application of eco-friendly ATPSs composed of water, ethyl lactate (EL), and organic salts, namely disodium succinate (Na_2_Succinate) and disodium tartrate (Na_2_Tartrate), for future application in the valorisation of food waste. All partitions presented successful results, with values of partition coefficients (*K*) higher than 1 and extraction efficiencies (*E*) higher than 50%, indicating a preferential migration of the polyphenols to the top phase. The extraction of FA using the ATPS based on Na_2_Tartrate presented the most promising results, with *K* = 19 ± 6 and *E* = (94.2 ± 0.9)% for the longest tie-line. Additionally, a comparison with previous works of the research group was drawn, with the extraction of RV exhibiting outstanding performance across all studied ATPSs. Therefore, the assessed ATPSs were shown to hold immense potential for the recovery of polyphenols.

## 1. Introduction

Every year, more than 1 billion tonnes of food are wasted globally, with losses occurring at various stages of the supply chain and representing over 30% of the world’s produced food [1]. Among the disposed materials may be non-edible parts, such as peels, stems, and seeds, as well as edible food waste resulting from spoilage or losses during production and processing. These different types of food waste could be repurposed for multiple applications, including energy generation, soil enrichment, and extraction of valuable substances like vitamins and antioxidants [2,3,4,5]. The recovery of these biomolecules, in particular, has been a very active focus of research, with several methods recently being explored for the effective extraction of these target species [6,7,8,9]. Additionally, it promotes the circular economy model by encouraging the responsible and sustainable use of resources and proper recycling of waste [10,11]. Therefore, the development of refined and sustainable extraction techniques is critical to tackling the issue of food waste.

One of the most promising methods for the extraction of bioactive substances is aqueous two-phase systems (ATPSs). This liquid–liquid extraction technique consists of a system composed of water and an additional component, to which a salting-out agent is added, inducing the formation of two distinct phases. The biomolecule then partitions between the phases, according to its hydrophobicity, polarity, and charge [12]. With both phases being mostly composed of water, ATPSs provide a bio-compatible medium that protects the integrity of the target species [13]. Being a simple, easy-to-scale-up, and cost-effective technique [14], ATPSs have been gaining significant attention, standing out among other common extraction techniques. Additionally, given the presence of bioactive substances in various types of food waste, ATPSs offer broad applicability in this field of research. Nevertheless, depending on the specific waste being treated, a pre-processing step may be required.

ATPSs can be composed of, for example, two polymers [15], a polymer, and a salt [16], or an ionic liquid and a salt [17]. However, with the need for greener alternatives, the implementation of organic solvents that are biodegradable and non-toxic for both humans and the environment has increasingly been studied. Ethyl lactate (EL) is an organic solvent that can easily be obtained through the fermentation of corn [18] and has been successfully employed in extractions using ATPSs [18,19,20,21], supporting its choice as a solvent for the ATPSs in the present work.

Moreover, the selection of salting-out agents is also crucial to achieving phase separation and enabling effective biomolecule extraction. Inorganic salts, such as phosphates and sulphates, have been extensively studied in literature [18,22,23], but their discharge at high concentrations raises significant environmental concerns [24,25]. As a result, alternative eco-friendly salting-out agents should be considered. Organic salts stand out as a promising alternative due to their strong potential as salting-out agents, along with their sustainable nature [26,27]. In addition, organic salts present a wide range of applications, including food preservation [28,29], the production of photovoltaic devices [30,31], and catalysis [32,33], which supports their subsequent recovery and repurpose. Considering the benefits mentioned, two organic salts, namely disodium succinate (Na_2_Succinate) and disodium tartrate (Na_2_Tartrate), have been employed in the studied ATPSs.

Among the biomolecules that can be extracted, polyphenols are particularly remarkable due to their wide range of health benefits, especially in the treatment of chronic diseases [34,35]. Notably, chlorogenic acid (CA), ferulic acid (FA), and resveratrol (RV) exhibit strong bioactive potential and are prevalent in the human diet.

Chlorogenic acid (CA), an ester of caffeic and quinic acids, is a polyphenol with the chemical structure of 5-caffeoylquinic acid [36]. It is mostly present in coffee beans, apples, sunflower seeds, and various berries [37]. This biomolecule has strong antioxidant and anti-inflammatory properties, playing a significant role in the protection against cardiovascular diseases and type II diabetes [38]. Furthermore, several studies indicate that the intake of this polyphenol may be helpful in the treatment of neurodegenerative conditions, such as Alzheimer’s and Parkinson’s diseases [39,40].

Ferulic acid (FA) is a phenolic antioxidant with the chemical structure of 4-hydroxy-3-methoxycinnamic acid. This compound is present in a wide variety of plant tissues, where it plays a key role in the plant cell walls, providing rigidity and aiding in the formation of various important organic compounds [41]. Beyond its function in plant physiology, it is also an important dietary compound for human health, commonly found in vegetables, cereal grains, and fruit. Considering its anti-inflammatory, anti-bacterial, and antioxidant effects [42], FA has been vastly used in the medical field, with a particular emphasis on protection against ultraviolet radiation [43] and the treatment of cognitive disorders [44].

Resveratrol (RV), or 3,4′,5-trihydroxystilbene, is a polyphenol synthesised by several types of legumes or berry-producing plants, such as grapes, peanuts, and blueberries, in response to injury or pathogenic attacks [45]. RV exhibits multiple bioactive effects, such as antioxidant, anti-inflammatory, anticarcinogenic, and cardioprotective effects, highlighting its vast potential in the treatment of various health conditions, including cancer [46] and cardiovascular diseases [47].

In this work, the extraction of CA, FA, and RV was conducted in two organic salt-based ATPSs, {Ethyl lactate (1) + Na_2_Succinate or Na_2_Tartrate (2) + Water (3)}, at 298.15 K and 0.1 MPa. This study aimed at making advancements in the application of food waste valorisation techniques, with particular focus on ATPSs. Furthermore, this method holds potential for future applications, including the production of food supplements and medicine.

## 2. Materials and Methods

### 2.1. Chemicals

Table 1 lists the chemicals used in this work, as well as their respective chemical formulae, supplying companies, purities, Chemical Abstracts Service (CAS) numbers, and abbreviations. These materials were used as provided by the suppliers, with no additional purification steps being conducted.

### 2.2. Equipment

Mass $\left(m\right)$ was measured with an Adam Equipment AAA 250L balance with a standard measurement uncertainty of 10^−4^ g, while pH was quantified using a VWR pH 1100L (Radnor, PA, USA) device with standard measurement uncertainties of 0.01 in pH and 0.1 K in temperature (T). The electrical conductivity $\left(\kappa \right)\;$of pure water was obtained with a Hanna EDGE EC conductivity meter, coupled with an HI763100 platinum conductivity cell with a relative measurement uncertainty of 0.01. In order to verify the water content of the organic salts and polyphenols used, a Mettler Toledo C20 Coulometric Karl Fisher titrator with a standard measurement uncertainty of 5 ppm was used. Hydrate water was accounted for during solution preparation. To regulate the temperature of the experiments, an OVAN Therm H TH100E thermostatic bath with a standard measurement uncertainty of 0.1 K was employed, with the temperature being checked with a glass thermometer with a standard measurement uncertainty of 0.01 K. For the UV-Vis absorbance measurements, a ThermoScientific Varioskan Flash ultraviolet–visible (UV-Vis) spectrophotometer was used, with a standard measurement uncertainty of 10^−4^. Liquid density $\left(\rho \right)\;$was measured using an Anton Paar DSA-4500M oscillating densimeter, with standard measurement uncertainties of 3 × 10^−5^ g·cm^−3^ in liquid density and 0.01 K in temperature. Before any experiments were conducted, the calibration of both the pH meter and the densimeter was carried out according to their respective manuals.

### 2.3. Experimental Procedure

#### 2.3.1. Influence of pH on the UV-Vis Absorbance Spectra

Changes in pH can lead to structure and charge modifications in the studied biomolecules due to protonation reactions (release of H^+^), affecting the mean electrical charge (q) of the target species [48,49]. This results in interaction changes that may affect the UV-Vis absorbance spectra of the biomolecules, as well as their partitioning. Therefore, a thorough study must be conducted on the effect of pH on the species.

The relative fraction ($x_{A}$) of ionised species, referred to as “stages” in this work [50], must be calculated. From the definition of the acid dissociation constant ($K_{a}$) at a set pH, the negative base-10 logarithm of the acid dissociation constants ($pK_{a}$) of each biomolecule can be related to the concentration ratios of different stages, as seen in Equation (1). Based on this expression, $x_{A}$ can be determined, as shown by Equation (2) [50].

(1) $$\;\frac{\left[A^{q_{0}-i+1}\right]}{\left[A^{q_{0}-i}\right]}=10^{pH_{phase}-{pK}_{a}^{i}}$$

(2) $$x_{A^{q_{0}-i+1}}=\frac{\left[A^{q_{0}-i+1}\right]}{\left[A^{q_{0}-1}\right]}/\left(\frac{\left[A^{q_{0}}\right]}{\left[A^{q_{0}-1}\right]}+1+\sum_{j=2}^{i_{max}}\left[\prod_{k=2}^{j}\frac{\left[A^{q_{0}-k}\right]}{\left[A^{q_{0}-\left(k-1\right)}\right]}\right]\right)$$

where $q_{0}$ is the electrical charge of the biomolecule A at pH=0; i is the number of donated protons; $\left[A^{q_{0}-i+1}\right]$ and $\left[A^{q_{0}-i}\right]$ are the mole concentrations of the stages, with electrical charges equal to $\left(q_{0}-i+1\right)\;e$ and $\left(q_{0}-i\right)\;e$, respectively, with e referring to the elementary charge (1.602 × 10^−19^ C); $x_{A^{q_{0}-i+1}}$ is the fraction of species with electrical charge equal to $\left(q_{0}-i+1\right)\;e$; and $i_{max}\;$is the maximum number of protons that can be donated.

From Equation (2), the mean electrical charge $\left(q\right)\;$of the biomolecules can be calculated using a weighted arithmetic mean, as Equation (3) shows.

(3) $$\;q=\sum_{i=1}^{i_{max}}\left[x_{A^{q_{0}-i+1}}\cdot \left(q_{0}-i+1\right)\right]+\left[1-\sum_{i=1}^{i_{max}}\left(x_{A^{q_{0}-i+1}}\right)\right]\cdot \left(q_{0}-i_{max}\right)$$

From the $pK_{a}$ values of the biomolecules, the relation between the mean electrical charge and pH could be determined, and specific pH values could be chosen to characterise each stage. With this information, aqueous stock solutions of chlorogenic acid (CA), ferulic acid (FA), and resveratrol (RV) were prepared with concentrations of 1.44 × 10^−4^, 8.25 × 10^−5^, and 6.66 × 10^−5^% in mass, respectively. The pH was then adjusted to the intended values by gradually adding droplets of 0.5 M aqueous solutions of acetic acid (CH_3_COOH) or sodium hydroxide (NaOH). Afterwards, the UV-Vis absorbance spectra were measured from 200 to 600 nm and at 298.15 K. Since the solutions were diluted through the addition of pH adjusters, Equation (4) was applied to normalise the obtained spectra, with this approximation being valid for minimal concentration differences. Finally, to determine whether the spectra were stable for the duration of the work (about 24 h), a new measurement was carried out after 3 days. This procedure had already been performed in previous studies for all three biomolecules [51,52,53], which were all shown to be stable for the pH of the ATPSs in the study.

(4) $$\;A^{\prime }=A\cdot \frac{C_{pH\approx 7}}{C_{pH=k}}$$

where A and A’ are the measured and normalised UV-Vis absorbances, respectively; $C_{pH\approx 7}$ is the reference concentration (chosen according to the characteristic pH of the studied ATPSs, which is approximately 7); and $C_{pH=k}$ is the concentration of the biomolecule solution at pH=k.

#### 2.3.2. Liquid–Liquid Equilibria Data

The tie-line composition for the disodium succinate (Na_2_Succinate) [20] and disodium tartrate (Na_2_Tartrate) [19] ATPSs was determined in previous works of the research group, with the data being shown in Table 2. In addition, the chemical structures of the two organic salts, as well as the phase equilibria diagrams with the feed compositions used for these systems, are displayed in Figures S1 and S2, respectively, in the Supplementary Materials.

#### 2.3.3. Biomolecule Extraction

To enable biomolecule quantification, UV-Vis calibration curves were employed. These had already been determined and validated in previous works involving the studied biomolecules [51,52,53]. Afterwards, and using the LLE data for each ATPS, experimental assays were conducted.

Using the mass composition of the tie-lines shown in Table 2, assays of 10 mL were prepared by pipetting ethyl lactate (EL), water, and binary aqueous solutions of organic salts (Na_2_Succinate: 23.06% in mass; Na_2_Tartrate: 26.87% in mass) using an Eppendorf Multipipette E3x electronic pipet. These assays were referred to as blanks. Following the addition of the components, the mixtures were stirred for 6 h and left to settle for 12 h at 298.15 K and 0.1 MPa. After this time, when equilibrium was achieved, the two distinct phases formed were subsequently separated. The mass, liquid density, pH, and UV-Vis absorbance of each phase were then measured.

Afterwards, a similar set of assays, referred to as partitions, were prepared. These constituted the same components as the blanks, except for 1 mL of water, which was substituted by 1 mL of biomolecule solution. To assure proper quantification, the biomolecule solutions used in this part of the work were of concentrations two to four times higher than the ones used in the pH study (CA: 4.05 × 10^−4^ % in mass; FA: 2.56 × 10^−4^ % in mass; RV: 9.19 × 10^−5^ % in mass). The partitions were then submitted to the same procedure as the blanks.

Finally, the absorbance of the blanks was subtracted from the partitions to obtain the biomolecule absorbance. It must be noted that, within this experiment, and following previous works on this topic [51,54,55], the influence of the biomolecule in ATPS phase composition is considered to be negligible, with the relative composition between the top and bottom phase remaining unchanged.

The biomolecule partitioning was evaluated through the analysis of performance indicators, namely the partition coefficient $\left(K\right)\;$and the extraction efficiency (E). The partition coefficient $\left(K\right)\;$is a measure of the solute distribution between the different phases, which is determined by the ratio between the biomolecule concentration in the top and bottom phases, as seen in Equation (5).

(5) $$\;K_{i}=\frac{C_{T,i}}{C_{B,i}}$$

where i is the tie-line number, C is the biomolecule concentration (in g·mL^−1^), and T and B refer to the top and bottom phases, respectively. The concentrations were calculated using the formerly determined calibration curves and the measured UV-Vis absorbances.

The extraction efficiency (E) indicates the percentage of solute that migrated to the top phase for each tie-line. This parameter, calculated through Equation (6), is given by the ratio between the mass of the biomolecule in the top phase and the feed mass of the biomolecule.

(6) $$\;E_{i}=\frac{C_{T,i}V_{T,i}}{m_{SF,i}}\times 100$$

where $m_{SF}$ is the feed mass of the biomolecule and V is the phase volume, which was determined using Equation (7).

(7) $$\;V_{f,i}=\frac{m_{f,i}}{\rho_{f,i}}$$

where f refers to the top (T) or bottom (B) phase, m is the phase mass, and ρ is the liquid density of the phase. It is crucial to analyse both performance indicators to better understand the acquired results. Particularly, in cases of significant differences in phase size, the analysis of a single indicator may result in an erroneous assessment of the extraction assays.

Finally, the mass losses in phase separation ($L_{m}$) and in quantification ($L_{s}$) were calculated through Equations (8) and (9), respectively, to validate the employed method.

(8) $$\;L_{m,i}=\frac{\left(m_{T,i}+m_{B,i}\right)-m_{F,i}}{m_{F,i}}\times 100$$

where i is the tie-line number, $m_{F}$ refers to the feed mass, and $m_{T}$ and $m_{B}$ refer to the mass of the top and bottom phases, respectively.

(9) $$\;L_{s,i}=\frac{m_{SQ,i}-m_{SF,i}}{m_{SF,i}}\times 100$$

where $m_{SF}$ and $m_{SQ}$ are the feed and quantified masses of the biomolecule. In turn, the quantified mass was calculated using Equation (10).

(10) $$\;m_{SQ,i}=C_{T,i}V_{T,i}+C_{B,i}V_{B,i}$$

where C is the biomolecule concentration (in g·mL^−1^), V is the phase volume, and T and B refer to the top and bottom phases, respectively.

Following the assessment of biomolecule partitioning in the studied ATPSs, the obtained results were compared with previous works of the research group focused on disodium succinate (Na_2_Succinate), disodium tartrate (Na_2_Tartrate), sodium potassium tartrate (NaKTartrate), tripotassium citrate (K_3_Citrate) and trisodium citrate (Na_3_Citrate), so as to identify the most effective systems for the extraction of CA, FA, and RV.

## 3. Results and Discussion

### 3.1. Influence of pH on the UV-Vis Absorbance Spectra

**Figure 1:**
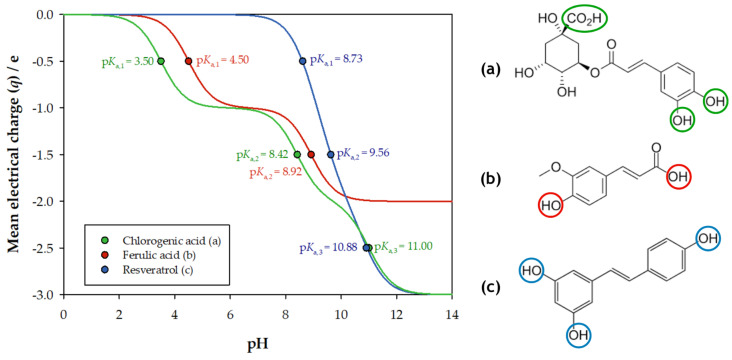
Calculated mean electrical charges with pH and chemical structure of chlorogenic acid (CA, (**a**)), ferulic acid (FA, (**b**)), and resveratrol (RV, (**c**)).

From Equations (1) to (3), and using the $pK_{a}$ values of the studied biomolecules (CA: 3.50, 8.42, and 11.00 [56]; FA: 4.50 and 8.92 [57]; RV: 8.73, 9.56, and 10.88 [58]), the relation between the mean electrical charge and pH could be determined. Figure 1 displays the graphical representation of this relation, as well as the chemical structure of each biomolecule, with the functional groups responsible for proton donation being outlined. Additionally, in Tables S1–S3 in the Supplementary Materials, the variation in stage fractions with pH for each biomolecule can be seen.

Chlorogenic acid (CA) has three $pK_{a}$ values, corresponding to the proton donation from the carboxylic acid group and the two phenolic groups. Ferulic acid (FA) contains one carboxylic acid group and one phenolic group, which is consistent with its two $pK_{a}$ values. Finally, resveratrol (RV) has three $pK_{a}$ values, associated with its three phenolic groups.

It can be observed that the biomolecules exhibit approximately integer mean electrical charges for neutral pH values (CA: −1.04 e; FA: −1.01 e; RV: −0.02 e). This indicates that only one stage is primarily present in the solution, which is favourable for the conducted work, as a mixture of species could have resulted in undesirable results. This is particularly evident in the analysis of the UV-Vis absorbance spectra, as chemical and structural modifications originating from proton loss may alter the characteristic spectra of the biomolecules. Therefore, the analysis of spectra changes and stability with pH is also crucial to the present study. This was assessed in previous works of the research group [51,52,53], with all the spectra being stable for the characteristic pH of the studied ATPSs (pH ≈ 7).

### 3.2. Biomolecule Extraction

The extraction of biomolecules was carried out at the lab scale with the primary objective of identifying the most effective systems for recovering each target species. This approach is a crucial initial step for the optimization of extraction, ensuring an increased success rate when scaled up to real biomolecule-rich substrates in future applications.

Following the procedure described in Section 2.3.3, the partition assays were conducted and the mass (m), liquid density (ρ), and pH of each phase were measured. Furthermore, Equation (9) was applied to determine mass losses in phase separation $\left(L_{m}\right)$. These results are displayed in Table 3.

As anticipated, the liquid density of the bottom phase was consistently higher than the top phase. Furthermore, the mass losses in phase separation were negligible, with values lower than 1% being observed. In what concerns pH, close to neutral values were measured, as is characteristic for the ATPSs in this study. With the measured properties being validated, the mass fractions of the biomolecules ($w_{biomolecule}$) were determined through the respective calibration curves. Afterwards, the mass losses in quantification ($L_{s}$), the partition coefficients (K), and the extraction efficiencies (E) could be calculated through the equations presented in Section 2.3.3. These results are exhibited in Table 4.

**Figure 2:**
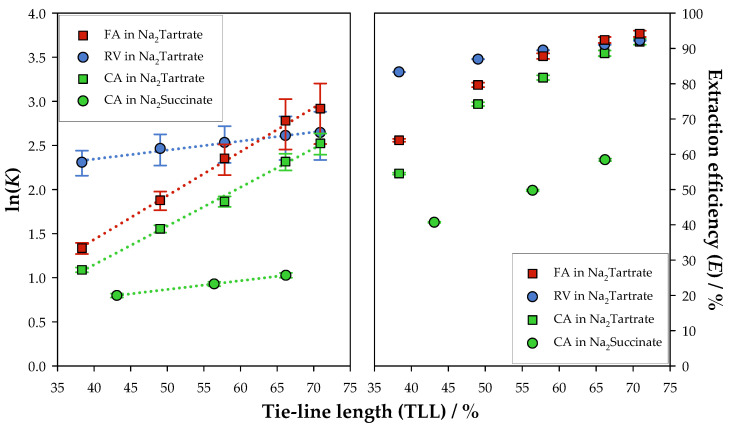
Extraction efficiencies (E) and natural logarithm of the partition coefficients (K) with tie-line length (TLL) for the extraction of chlorogenic acid (CA), ferulic acid (FA), and resveratrol (RV) in the ATPSs {Ethyl lactate (1) + Na_2_Succinate or Na_2_Tartrate (2) + Water (3)}, at 298.15 K and 0.1 MPa.

The mass losses in quantification ($L_{s}$) were consistently lower than 5%, which validates UV-Vis absorbance as a proper quantification method. Moreover, the biomolecule concentration was always higher in the top phase, which is indicative of a successful extraction. Nevertheless, the evaluation of the extraction must be conducted through the analysis of the performance indicators, partition coefficient (K), and extraction efficiency (E). In order to better understand and compare these performance indicators, a graphical representation of their relationship with tie-line length is presented in Figure 2.

All systems presented successful extraction results, with values of K>1 and E>50%, indicating that the majority of the biomolecule migrated to the top phase. These results can be attributed to the salting-out mechanism of the organic salts, which reduces the solubility of the biomolecules in water, leading to their migration from the water-rich phase (bottom) to the EL-rich phase (top). Moreover, an increase in the performance indicators was observed for longer tie-lines. As shown in Figure S2 in the Supplementary Materials, longer tie-lines correspond to higher concentrations of EL in the top phase and higher concentrations of organic salt in the bottom phase. Therefore, the increase in the concentration of these components enhances the salting-out effect, favouring a preferential migration of the target species to the top phase. On the other hand, the affinity between the polyphenols and the components in the solution might also affect the partitioning of the biomolecules, as less polar polyphenols might have a higher affinity for EL than water, migrating more easily to the top phase.

Among the studied partitions, the ATPS based on Na_2_Succinate, compared to the ATPS based on Na_2_Tartrate, presented significantly lower values of both K and E, translating to a lower performance of extraction, achieving the maximum values of 2.79 ± 0.08 for the partition coefficient and (58.4 ± 0.4)% for the extraction efficiency. To assess the performance differences between the two salts, their chemical structures (shown in Figure S1 of the Supplementary Materials) were compared. Na_2_Tartrate contains two additional hydroxyl groups compared to Na_2_Succinate, which may increase its affinity to water and enhance the salting-out effect, leading to a more effective extraction of the polyphenol. Nevertheless, the mechanisms behind partitioning are extremely complex, and variables such as the hydrophobicity of the components, the salting-out strength of the salts, and the biospecific affinity of the target species are known to influence the extraction process [59]. Therefore, each case must be studied individually to identify the key factors that influence biomolecule partitioning.

Within the systems based on Na_2_Tartrate, all biomolecules presented notably high partition coefficients and extraction efficiencies, with values over 10 and 90%, respectively, for the longest tie-line. Considering that EL is less polar than water, less polar polyphenols, such as RV, are expected to present higher performance indicators than more polar ones, such as CA. However, this is only verified for lower TLLs. For longer tie-lines, FA presented the best results, achieving a maximum partition coefficient of 19 ± 6 and an extraction efficiency of (94.2 ± 0.9)% for the longest tie-line. As a result, the increase in the concentration of EL in the top phase and of the salt in the bottom phase altered the performance order, likely due to the enhancement of the salting-out effect, which was more pronounced in FA than in RV.

Between CA and RV, although RV presented higher values for the performance indicators across the studied tie-line lengths, there seemed to be a tendency for the partition coefficients of CA to increase for longer tie-line lengths. Even so, as it is not possible to extrapolate data, RV was better extracted through the ATPS based on Na_2_Tartrate.

**Figure 3:**
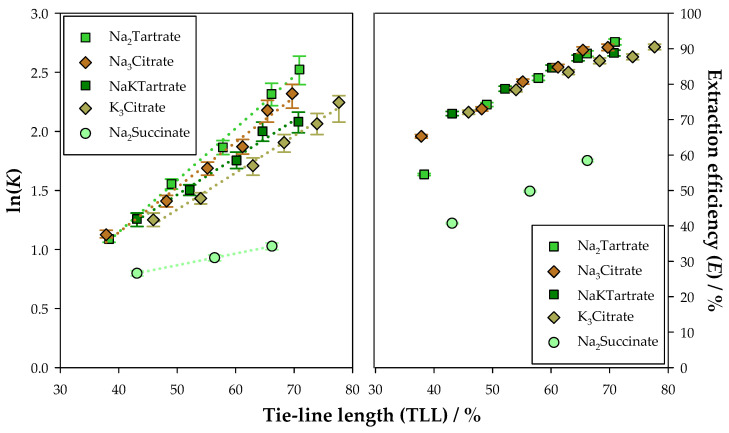
Natural logarithm of the partition coefficients (K) and extraction efficiencies (E) with tie-line length (TLL) for the extraction of chlorogenic acid (CA) in the ATPSs {Ethyl lactate (1) + Na_2_Succinate or Na_2_Tartrate or NaKTartrate [51] or Na_3_Citrate [51] or K_3_Citrate [51] (2) + Water (3)}, at 298.15 K and 0.1 MPa.

Given that the biomolecules used in this work have been widely studied in the research group, a comparison with previously obtained results could be carried out. Initially, the results obtained for CA were compared in ATPSs based on Na_2_Succinate, Na_2_Tartrate, NaKTartrate, Na_3_Citrate, and K_3_Citrate, as seen in Figure 3. Moreover, the graphics represented in Figure 3 are depicted separately in Figures S3 and S4 in the Supplementary Materials for easier visualisation.

By comparing the extraction of CA using the various ATPSs, it could be observed that the ATPS based on Na_2_Succinate presented the poorest performance among the studied ATPSs, with considerably lower values for both K and E. Succinate salts, being less hydrophilic than tartrate and citrate salts, present the lowest salting-out effect. Therefore, as observed with similar works in the literature [60,61], the use of a weaker salting-out agent leads to a worse extraction and, consequently, lower partition coefficients and extraction efficiencies. This is even more prevalent in the case of less hydrophobic biomolecules such as CA, which have a higher affinity to water and are more heavily influenced by the salting-out effect.

Considering tartrate and citrate salts have a stronger salting-out effect, better extraction results were obtained for these systems, with the ATPS based on Na_2_Tartrate presenting the best performance. As this study is the first to extract CA using the ATPS based on Na_2_Tartrate, it allowed for the identification of a more effective approach for the recovery of this polyphenol, highlighting the significance of the conducted work.

**Figure 4:**
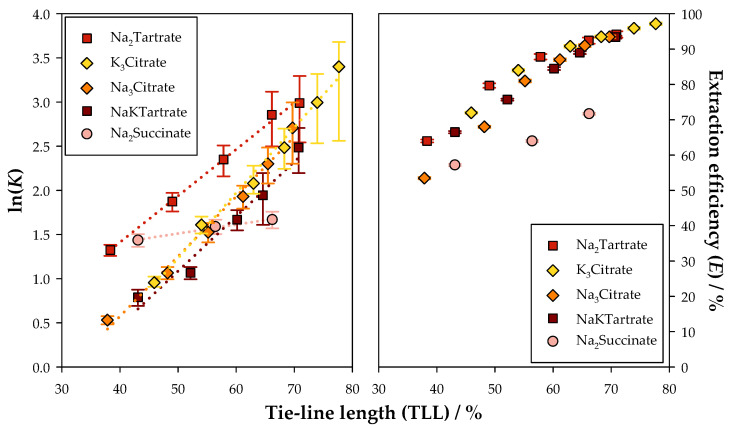
Natural logarithm of the partition coefficients (K) and extraction efficiencies (E) with tie-line length (TLL) for the extraction of ferulic acid (FA) in the ATPSs {Ethyl lactate (1) + Na_2_Succinate [62] or Na_2_Tartrate or NaKTartrate [62] or Na_3_Citrate [52] or K_3_Citrate [52] (2) + Water (3)}, at 298.15 K and 0.1 MPa.

Afterwards, a comparison was drawn for the extraction of FA using ATPSs based on Na_2_Tartrate, Na_2_Succinate, NaKTartrate, Na_3_Citrate, and K_3_Citrate. The results are illustrated in Figure 4. Additionally, the graphics shown in Figure 4 are presented separately and in Figures S5 and S6 in the Supplementary Materials.

As observed with CA, the Na_2_Succinate system presented the lowest partition coefficients and extraction efficiencies, displaying lower extraction performance. However, the disparity among the different salt-based systems was much less pronounced, which might be associated with the lower hydrophilicity of this polyphenol and, consequently, higher affinity to the top phase.

In what concerns the citrate and tartrate salt-based systems, the ATPS based on K_3_Citrate exhibited the highest values of K and E for the longest tie-line. Nevertheless, when comparing the same tie-line length, the Na_2_Tartrate system always provided better results, and therefore is considered the best ATPS for the retrieval of FA. Regardless, these two systems presented considerably high values for both performance indicators, pointing to an extremely effective extraction.

**Figure 5:**
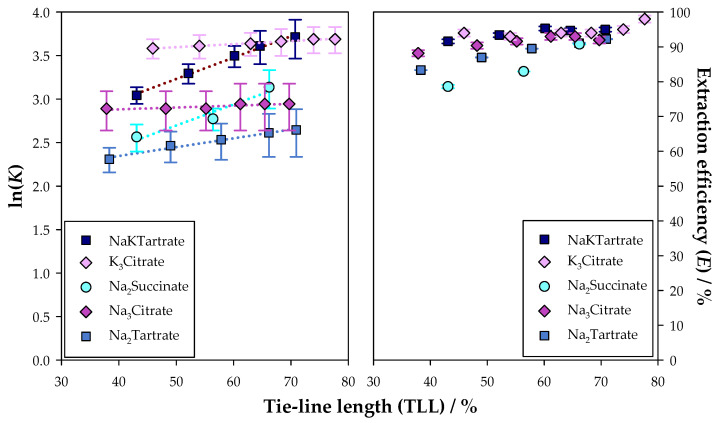
Natural logarithm of the partition coefficients (K) and extraction efficiencies (E) with tie-line length (TLL) for the extraction of resveratrol (RV) in the ATPSs {Ethyl lactate (1) + Na_2_Succinate [53] or Na_2_Tartrate or NaKTartrate [53] or Na_3_Citrate [53] or K_3_Citrate [53] (2) + Water (3)}, at 298.15 K and 0.1 MPa.

Finally, a comparison of the recovery of RV in Na_2_Tartrate, Na_2_Succinate, NaKTartrate, Na_3_Citrate, and K_3_Citrate ATPSs was conducted, with the results being shown in Figure 5. In addition, in Figures S7 and S8 in the Supplementary Materials, the graphics shown in Figure 5 are presented separately to ease interpretation.

In general, the performance indicators of RV were seen to be higher in comparison to CA and FA, all with values of K>10 and E>90%, for the longest tie-lines. Considering the lower polarity of RV and its consequently higher affinity to EL than to water, this polyphenol was expected to migrate preferentially to the top phase. Moreover, its partitioning appears to have been driven more by the affinity between the components than by the salting-out effect, as indicated by the lower slopes of the partition coefficients for RV, which imply a lower influence of salt concentration.

When analysing the partition coefficients, some differences could be noted between the studied systems. For lower TLLs, the following order was observed: K_3_Citrate > NaKTartrate > Na_3_Citrate > Na_2_Succinate > Na_2_Tartrate. Potassium-containing salts were seen to lead to better extraction results in comparison to sodium-containing ones. Additionally, within these two groups of salts, citrates presented higher partition coefficients than tartrates. However, for longer tie-lines, there seemed to be a change in the order of extraction performance, with Na_2_Succinate and NaKTartrate displaying a higher partition coefficient than K_3_Citrate and Na_3_Citrate, respectively. Thus, the increase in salt concentration resulted in a stronger salting-out power, leading to higher partition coefficients for both succinate and tartrate salts. This makes it difficult to determine which system is superior, as it is dependent on the extraction conditions. Regardless, both NaKTartrate and K_3_Citrate ATPSs presented outstanding results in the recovery of RV, with higher partition coefficients than in the remaining biomolecules.

Taking into account the obtained data and the conducted comparison of results, a general analysis of the performance of the ATPSs in the extraction of the polyphenols can be carried out. The ATPS based on Na_2_Succinate presented the weakest performance overall, with generally lower partition coefficients and extraction efficiencies than the remaining systems. In contrast, the other ATPSs displayed broad variation in performance, depending on the extracted target species. Moreover, all biomolecules were seen to be effectively extracted through the assessed ATPSs, with RV yielding the most promising results, exhibiting considerable high-performance indicators across all ATPSs.

## 4. Conclusions

In this work, the extraction of three polyphenols, namely chlorogenic acid (CA), ferulic acid (FA), and resveratrol (RV), was conducted using the aqueous two-phase systems (ATPSs) {Ethyl lactate (1) + Na_2_Succinate or Na_2_Tartrate (2) + Water (3)}, at 298.15 K and 0.1 MPa. All target species were successfully extracted, with partition coefficients higher than unity and extraction efficiencies above 50% being obtained, indicating a preferential migration of the biomolecules towards the top phase. This may have been due to the salting-out effect of the organic salts, which reduced the solubility of the biomolecules in water, promoting their migration to the EL-rich phase. Furthermore, the affinity between the components might also have affected partitioning, as less polar polyphenols might have migrated more easily to the top phase. Overall, the ATPS based on Na_2_Tartrate presented more promising results than the one based on Na_2_Succinate. FA exhibited the best results, with a maximum partition coefficient of 19 ± 6 and an extraction efficiency of (94.2 ± 0.9)% for the longest tie-line of the system containing Na_2_Tartrate.

Additionally, a comparison with previous works was conducted to determine which ATPSs were more effective in the recovery of each polyphenol, with CA and FA being more easily extracted by the ATPS based on Na_2_Tartrate, while RV was favoured by the ATPSs based on K_3_Citrate and NaKTartrate. Considering all the obtained results, RV was shown to be the most easily recovered biomolecule, consistently presenting high-performance indicators in all studied ATPSs.

## Figures and Tables

**Table 1:** List of chemicals, with the respective chemical formula, supplier, purity, CAS number, and abbreviation.

Chemical	Supplier	Purity/% ^a^	CAS	Abbreviation
Acetic acid ($C_{2}H_{4}O_{2}$)	Merck	>99.8	64-19-7	-
Chlorogenic acid ($C_{16}H_{18}O_{9}$)	Apollo Scientific	>98.0	327-97-9	CA
Disodium L(+)-tartrate dihydrate ($C_{4}H_{4}{Na}_{2}O_{6}{\cdot 2H}_{2}O$)	VWR Chemicals	>99.9	6106-24-7	Na_2_Tartrate
Disodium succinate hexahydrate ($C_{4}H_{4}{Na}_{2}O_{4}$·${6H}_{2}O$)	Tokyo Chemical Industry	>95.0	6106-21-4	Na_2_Succinate
Ethanol ($C_{2}H_{6}O$)	Sigma-Aldrich	>99.0	64-17-5	-
(-)-ethyl L-lactate ($C_{5}H_{10}O_{3}$)	Sigma-Aldrich	>98.0	97-64-3	EL
Ferulic acid ($C_{10}H_{10}O_{4}$)	Sigma-Aldrich	>99.0	537-98-4	FA
Purified water ($H_{2}O$)	VWR chemicals	^b^	7732-18-5	-
Resveratrol ($C_{14}H_{12}O_{3}$)	Tokyo Chemical Industry	>99.0	501-36-0	RV
Sodium hydroxide (NaOH)	Merck	>99.0	1310-73-2	-

^a^ Provided by the supplier in mass percentage. ^b^ The electrical conductivity $\left(\kappa \right)\;$of pure water was found to be 5 × 10^−6^ S·m^−1^.

**Table 2:** Tie-line compositions for the ATPSs {Ethyl lactate (EL) (1) + Na_2_Succinate [20] or Na_2_Tartrate [19] (2) + Water (3)}, at 298.15 K and 0.1 MPa ^a,b^.

No. TL	Feed		Top Phase			Bottom Phase			TLL/%
	$w_{EL,feed}$	$w_{salt,feed}$	$w_{EL,top}$	$w_{salt,top}$	pH	$w_{EL,bot}$	$w_{salt,bot}$	pH	
**{EL (1) + Na_2_Succinate (2) + Water (3)} [20]**									
TL1	0.280	0.126	0.602	0.030	7.25	0.189	0.153	7.25	43.10
TL2	0.300	0.130	0.687	0.019	7.45	0.145	0.174	7.37	56.40
TL3	0.320	0.135	0.742	0.014	7.37	0.106	0.196	7.34	66.20
**{EL (1) + Na_2_Tartrate (2) + Water (3)} [19]**									
TL1	0.280	0.125	0.514	0.037	6.18	0.155	0.170	6.10	38.33
TL2	0.300	0.130	0.575	0.027	6.13	0.116	0.198	6.17	49.02
TL3	0.325	0.133	0.631	0.020	6.13	0.090	0.223	6.18	57.82
TL4	0.355	0.138	0.689	0.015	6.15	0.070	0.248	6.18	66.14
TL5	0.380	0.140	0.724	0.013	6.11	0.060	0.262	6.17	70.90

^a^ $w_{i}\;$stands for the mass fraction of species i. ^b^ The reported standard measurement uncertainties (u) are $u(w_{i})$ = 10^−3^, u(pH) = 10^−2^, u(T) = 0.01 K, and u(P) = 2 kPa.

**Table 3:** Experimental phase mass (m), mass loss percentage in phase separation $\left(L_{m}\right)$, liquid density (ρ), and pH for the extraction of chlorogenic acid (CA), ferulic acid (FA), and resveratrol (RV) in the ATPSs {EL (1) + Na_2_Succinate or Na_2_Tartrate (2) + Water (3)}, at 298.15 K and 0.1 MPa ^a^.

Tie-Line	Phase	m/g	$L_{m}$/%	ρ/kg·m^−3^	pH
**CA in {EL (1) + Na_2_Succinate (2) + Water (3)}**					
1	Top	2.3287	−0.47	1051.09	6.89
	Bottom	7.6379		1119.96	7.05
2	Top	2.8385	−0.34	1047.78	7.26
	Bottom	7.1535		1133.28	7.09
3	Top	3.4070	−0.52	1047.92	7.21
	Bottom	6.5926		1144.22	7.09
**CA in {EL (1) + Na_2_Tartrate (2) + Water (3)}**					
1	Top	2.9069	−0.40	1058.71	5.87
	Bottom	7.0442		1128.28	5.81
2	Top	3.8477	−0.36	1051.34	5.78
	Bottom	6.1117		1149.91	5.86
3	Top	4.3057	−0.56	1049.28	5.75
	Bottom	5.6142		1166.07	5.92
4	Top	4.7111	−0.51	1045.32	5.93
	Bottom	5.1963		1189.52	5.87
5	Top	5.1467	−0.42	1045.99	5.86
	Bottom	4.8005		1197.33	5.90
**FA in {EL (1) + Na_2_Tartrate (2) + Water (3)}**					
1	Top	3.1550	−0.49	1059.54	5.72
	Bottom	6.7898		1129.98	5.91
2	Top	3.7805	−0.46	1053.15	5.84
	Bottom	6.1624		1150.08	5.84
3	Top	4.2549	−0.51	1048.09	5.80
	Bottom	5.6572		1167.13	5.82
4	Top	4.6796	−0.39	1045.49	5.74
	Bottom	5.2509		1189.72	5.84
5	Top	4.9603	−0.37	1044.86	5.93
	Bottom	4.9714		1200.81	5.93
**RV in {EL (1) + Na_2_Tartrate (2) + Water (3)}**					
1	Top	3.4302	−0.57	1059.76	5.99
	Bottom	6.4994		1130.21	5.97
2	Top	3.8123	−0.52	1053.09	5.87
	Bottom	6.1189		1147.94	5.99
3	Top	4.2850	−0.53	1048.92	5.74
	Bottom	5.6554		1165.93	5.96
4	Top	4.6785	−0.57	1045.44	5.91
	Bottom	5.2499		1188.93	6.11
5	Top	5.0137	−0.43	1044.30	5.96
	Bottom	4.9371		1205.12	6.09

^a^ The standard measurement uncertainties (u) are $u\left(m\right)=10^{-4}\;g$, $u\left(\rho \right)=0.03\;kg\cdot m^{-3}$, $u\left(pH\right)=10^{-2}$, $u\left(T\right)=0.01\;K$, and $u\left(P\right)=2\;kPa$.

**Table 4:** Tie-line length (TLL), mass fraction of biomolecule ($w_{biomolecule}$), mass loss percentage in quantification ($L_{s}$), partition coefficient (K), and extraction efficiency (E) for chlorogenic acid (CA), ferulic acid (FA), and resveratrol (RV) in the ATPSs {EL (1) + Na_2_Succinate or Na_2_Tartrate (2) + Water (3)}, at 298.15 K and 0.1 MPa ^a^.

Tie-Line	TLL/%	Phase	$w_{biomolecule}$	$L_{s}$/%	K	E/%
**CA in {EL (1) + Na_2_Succinate (2) + Water (3)}**						
1	43.10	Top	7.54 × 10^−5^	−2.86	2.22 ± 0.05	40.7 ± 0.2
		Bottom	3.40 × 10^−5^			
2	56.40	Top	7.59 × 10^−5^	−4.42	2.53 ± 0.07	49.8 ± 0.3
		Bottom	3.00 × 10^−5^			
3	66.20	Top	7.28 × 10^−5^	−4.50	2.79 ± 0.08	58.4 ± 0.4
		Bottom	2.60 × 10^−5^			
**CA in {EL (1) + Na_2_Tartrate (2) + Water (3)}**						
1	38.33	Top	8.12 × 10^−5^	−3.72	2.97 ± 0.08	54.5 ± 0.3
		Bottom	2.73 × 10^−5^			
2	49.02	Top	8.22 × 10^−5^	−3.00	4.7 ± 0.2	74.2 ± 0.6
		Bottom	1.74 × 10^−5^			
3	57.82	Top	7.99 × 10^−5^	−3.42	6.5 ± 0.4	81.7 ± 0.7
		Bottom	1.24 × 10^−5^			
4	66.14	Top	7.91 × 10^−5^	−2.90	10 ± 1	88.6 ± 0.8
		Bottom	7.80 × 10^−6^			
5	70.90	Top	7.62 × 10^−5^	−2.10	12 ± 2	91.9 ± 0.9
		Bottom	6.10 × 10^−6^			
**FA in {EL (1) + Na_2_Tartrate (2) + Water (3)}**						
1	38.33	Top	5.53 × 10^−5^	−2.02	3.8 ± 0.3	64.0 ± 0.5
		Bottom	1.46 × 10^−5^			
2	49.02	Top	5.65 × 10^−5^	−2.14	6.5 ± 0.7	79.7 ± 0.7
		Bottom	8.64 × 10^−6^			
3	57.82	Top	5.53 × 10^−5^	−2.19	11 ± 2	87.8 ± 0.8
		Bottom	5.26 × 10^−6^			
4	66.14	Top	5.28 × 10^−5^	−1.93	16 ± 5	92.4 ± 0.9
		Bottom	3.27 × 10^−6^			
5	70.90	Top	5.11 × 10^−5^	−1.40	19 ± 6	94.2 ± 0.9
		Bottom	2.76 × 10^−6^			
**RV in {EL (1) + Na_2_Tartrate (2) + Water (3)}**						
1	38.33	Top	2.37 × 10^−5^	−1.96	10 ± 2	83.34 ± 0.04
		Bottom	2.35 × 10^−6^			
2	49.02	Top	2.21 × 10^−5^	−2.16	12 ± 3	86.96 ± 0.04
		Bottom	1.88 × 10^−6^			
3	57.82	Top	2.03 × 10^−5^	−2.05	13 ± 3	89.51 ± 0.05
		Bottom	1.61 × 10^−6^			
4	66.14	Top	1.86 × 10^−5^	−2.35	14 ± 4	91.07 ± 0.05
		Bottom	1.36 × 10^−6^			
5	70.90	Top	1.75 × 10^−5^	−2.16	14 ± 4	92.26 ± 0.05
		Bottom	1.24 × 10^−6^			

^a^ The standard measurement uncertainties $\left(u\right)\;$are $u\left(T\right)=0.01\;K\;$and $u\left(P\right)=2\;kPa$.

## Data Availability

The data presented in this study are available on request from the corresponding authors.

## Supplementary Materials

The following Supplementary Materials can be downloaded at https://www.mdpi.com/article/10.3390/molecules30071532/s1: Figure S1: Chemical structures of disodium succinate (Na_2_Succinate) (a) and disodium tartrate (Na_2_Tartrate) (b); Figure S2: Liquid–liquid equilibria (LLE) phase diagrams with used feed compositions for the ATPSs {Ethyl lactate (1) + Na_2_Succinate or Na_2_Tartrate (2) + Water (3)} at 298.15 K and 0.1 MPa [20]; Figure S3: Natural logarithm of the partition coefficients (K) with tie-line length (TLL) for the extraction of chlorogenic acid (CA) in the ATPSs {Ethyl lactate (1) + Na_2_Succinate or Na_2_Tartrate or NaKTartrate or Na_3_Citrate or K_3_Citrate (2) + Water (3)} at 298.15 K and 0.1 MPa [19]; Figure S4: Extraction efficiencies (E) with tie-line length (TLL) for the extraction of chlorogenic acid (CA) in the ATPSs {Ethyl lactate (1) + Na_2_Succinate or Na_2_Tartrate or NaKTartrate or Na_3_Citrate or K_3_Citrate (2) + Water (3)} at 298.15 K and 0.1 MPa [51]; Figure S5: Natural logarithm of the partition coefficients (K) with tie-line length (TLL) for the extraction of ferulic acid (FA) in the ATPSs {Ethyl lactate (1) + Na_2_Succinate or Na_2_Tartrate or NaKTartrate or Na_3_Citrate or K_3_Citrate (2) + Water (3)} at 298.15 K and 0.1 MPa [52,62]; Figure S6: Extraction efficiencies (E) with tie-line length (TLL) for the extraction of ferulic acid (FA) in the ATPSs {Ethyl lactate (1) + Na_2_Succinate or Na_2_Tartrate or NaKTartrate or Na_3_Citrate or K_3_Citrate (2) + Water (3)} at 298.15 K and 0.1 MPa [52,62]; Figure S7: Natural logarithm of the partition coefficients (K) with tie-line length (TLL) for the extraction of resveratrol (RV) in the ATPSs {Ethyl lactate (1) + Na_2_Succinate or Na_2_Tartrate or NaKTartrate or Na_3_Citrate or K_3_Citrate (2) + Water (3)} at 298.15 K and 0.1 MPa [53]; Figure S8. Extraction efficiencies (E) with tie-line length (TLL) for the extraction of resveratrol (RV) in the ATPSs {Ethyl lactate (1) + Na_2_Succinate or Na_2_Tartrate or NaKTartrate or Na_3_Citrate or K_3_Citrate (2) + Water (3)} at 298.15 K and 0.1 MPa [53]; Table S1: Stage fraction and mean electrical charge (q) at different pH values for chlorogenic acid (CA), considering $pK_{a}$ values of 3.50, 8.42, and 11.00 [56]; Table S2: Stage fraction and mean electrical charge (q) at different pH values for ferulic acid (FA), considering ${pK}_{a}$ values of 4.50 and 8.92 [57]; Table S3: Stage fraction and mean electrical charge (q) at different pH values for resveratrol (RV), considering ${pK}_{a}$ values of 8.73, 9.56, and 10.88 [58].

## Abbreviations

The following abbreviations were used in this manuscript:

ATPSs	Aqueous two-phase systems
CA	Chlorogenic acid
EL	Ethyl lactate
FA	Ferulic acid
K_3_Citrate	Tripotassium citrate
LLE	Liquid–liquid equilibria
Na_3_Citrate	Trisodium citrate
Na_2_Succinate	Disodium succinate
Na_2_Tartrate	Disodium tartrate
NaKTartrate	Sodium potassium tartrate
RV	Resveratrol
TLL	Tie-line length
