# Supplementary material for: Retrieval of Polyphenols Using Aqueous Two-Phase Systems Based on Ethyl Lactate and Organic Salts
Source: Molecules. 2025 Mar 30;30(7):1532. doi: 10.3390/molecules30071532 (PMC11990810; doi:10.3390/molecules30071532)
Supplement: Supplementary file 1 [file molecules-30-01532-s001.zip › molecules-3547794-supplementary.pdf]

Supplementary Materials:

Retrieval of Polyphenols Using Aqueous Two-Phase Systems Based on Ethyl Lactate and Organic Salts

Gonçalo Perestrelo [1,2,a], Pedro Velho [1,2,b,*] and Eugénia A. Macedo [1,2,c,*]

[1] LSRE-LCM – Laboratory of Separation and Reaction Engineering - Laboratory of Catalysis and Materials, Faculty of Engineering, University of Porto, Rua Dr. Roberto Frias, 4200-465 Porto, Portugal.

[2] ALiCE – Associate Laboratory in Chemical Engineering, Faculty of Engineering, University of Porto, Rua Dr. Roberto Frias, 4200-465 Porto, Portugal.

[a] E-mail: gperestrelo@ fe.up.pt

[b] E-mail: velho@fe.up.pt

[c] E-mail: eamacedo@fe.up.pt

* Corresponding authors.

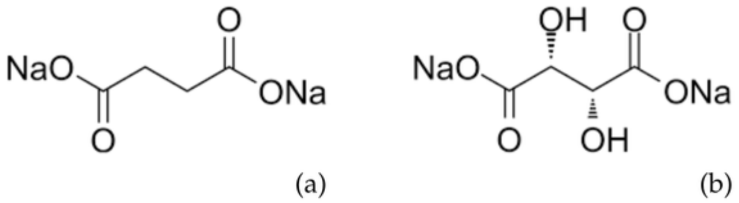

Figure S1. Chemical structures of disodium succinate (Na_2Succinate) (a) and disodium tartrate (Na_2Tartrate) (b).

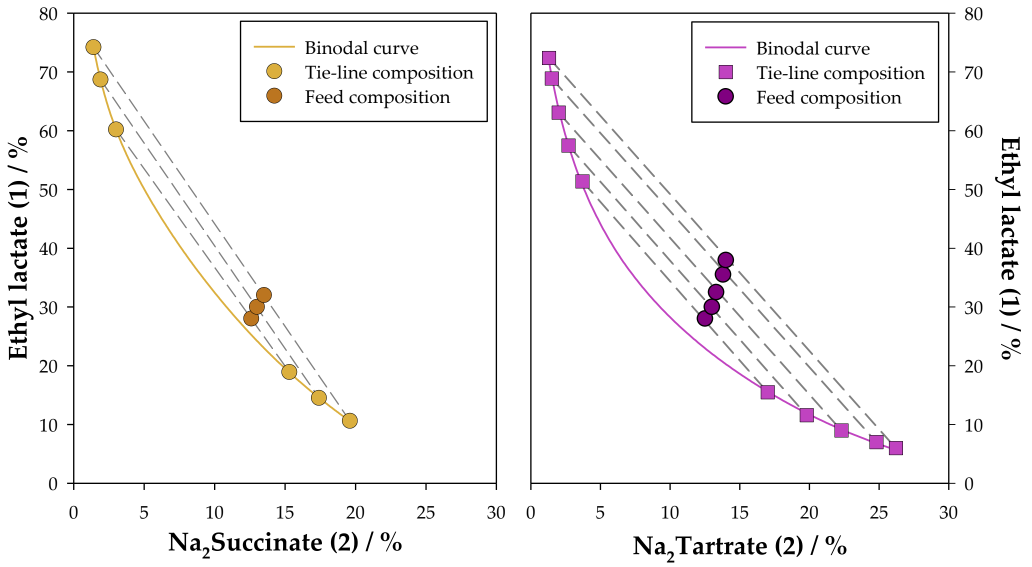

Figure S2. Liquid-liquid equilibria (LLE) phase diagrams with used feed compositions for the ATPSs {Ethyl lactate (1) + Na_2Succinate [1] or Na_2Tartrate [2] (2) + Water (3)}, at 298.15 K and 0.1 MPa.

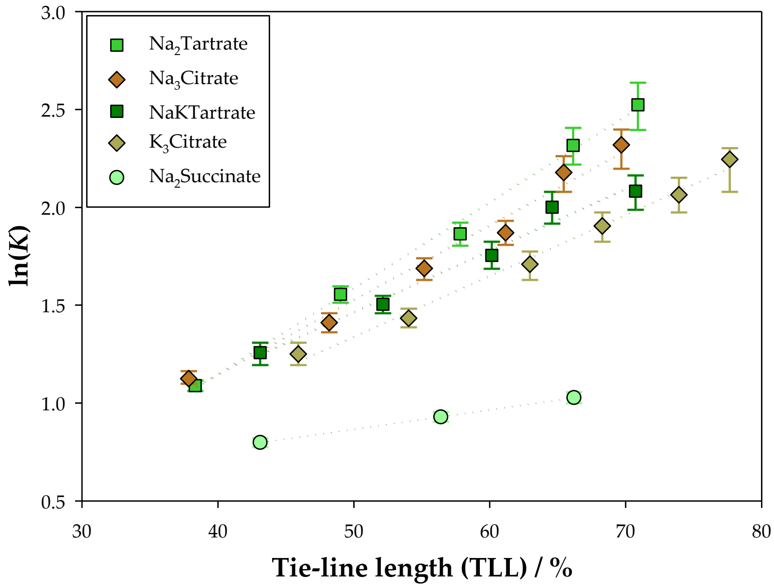

Figure S3. Natural logarithm of the partition coefficients (K) with tie-line length (TLL) for the extraction of chlorogenic acid (CA) in the ATPSs {Ethyl lactate (1) + Na_2Succinate or Na_2Tartrate or NaKTartrate [3] or Na_3Citrate [3] or K_3Citrate [3] (2) + Water (3)}, at 298.15 K and 0.1 MPa.

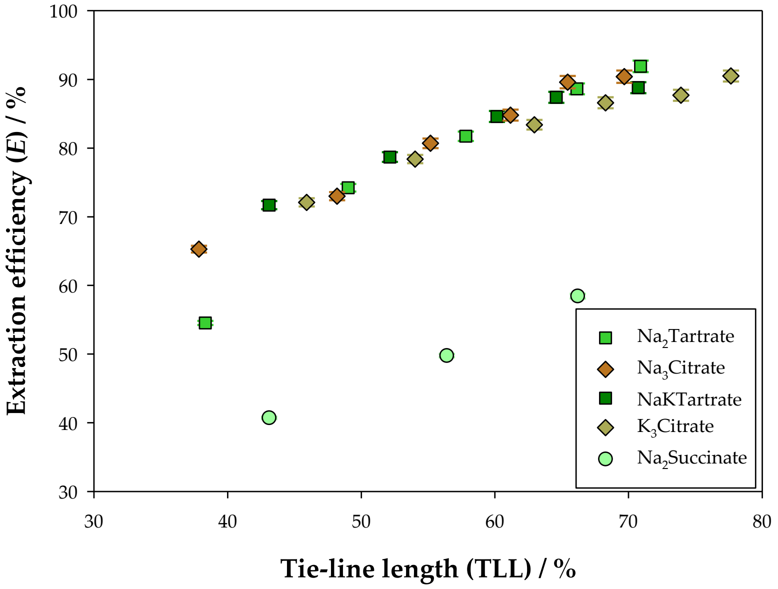

Figure S4. Extraction efficiencies (*E*) with tie-line length (TLL) for the extraction of chlorogenic acid (CA) in the ATPSs {Ethyl lactate (1) + Na_2Succinate or Na_2Tartrate or NaKTartrate [3] or Na_3Citrate [3] or K_3Citrate [3] (2) + Water (3)}, at 298.15 K and 0.1 MPa.

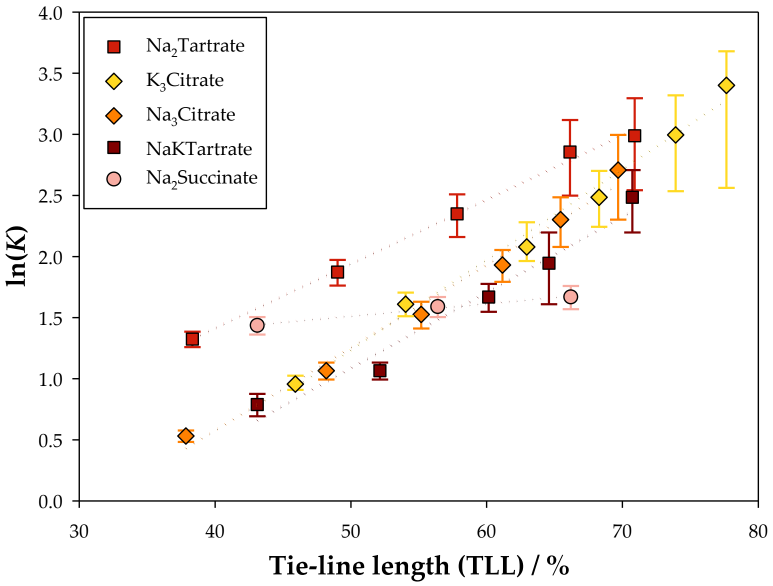

Figure S5. Natural logarithm of the partition coefficients (*K*) with tie-line length (TLL) for the extraction of ferulic acid (FA) in the ATPSs {Ethyl lactate (1) + Na_2Succinate [4] or Na_2Tartrate or NaKTartrate [4] or Na_3Citrate [5] or K_3Citrate [5] (2) + Water (3)}, at 298.15 K and 0.1 MPa.

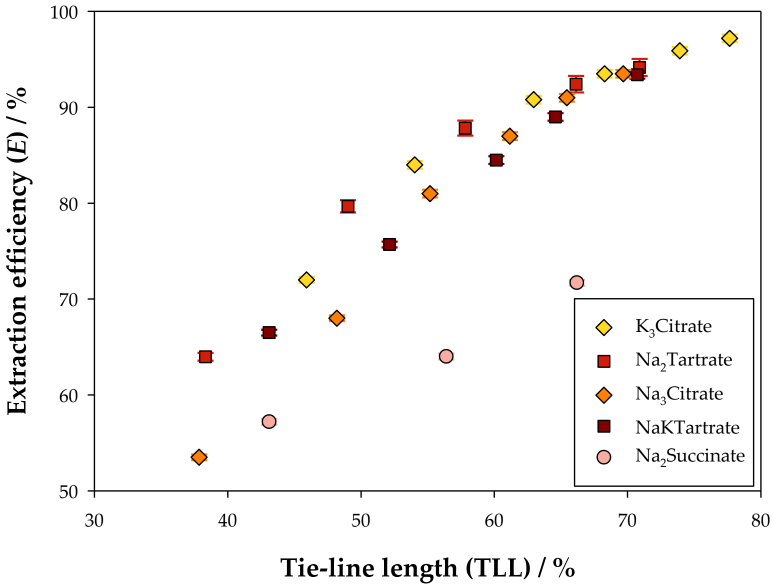

Figure S6. Extraction efficiencies (E) with tie-line length (TLL) for the extraction of ferulic acid (FA) in the ATPSs {Ethyl lactate (1) + Na_2Succinate [4] or Na_2Tartrate or NaKTartrate [4] or Na_3Citrate [5] or K_3Citrate [5] (2) + Water (3)}, at 298.15 K and 0.1 MPa.

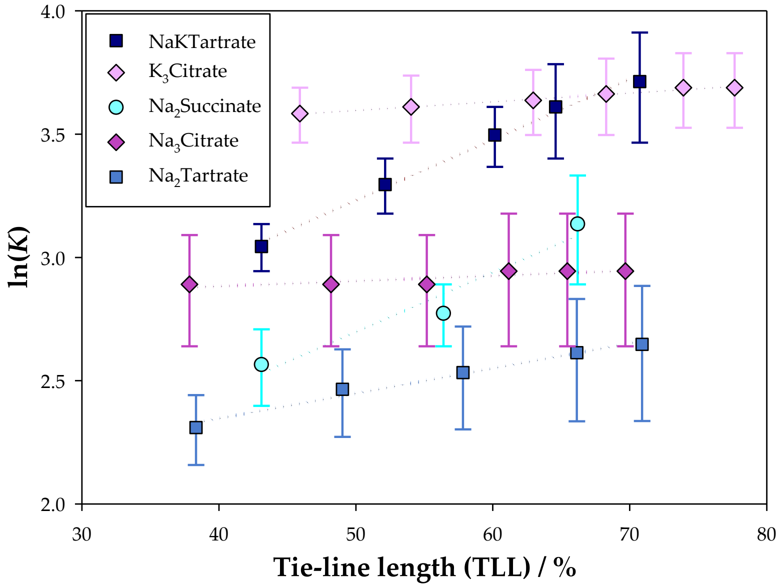

Figure S7. Natural logarithm of the partition coefficients (K) with tie-line length (TLL) for the extraction of resveratrol (RV) in the ATPSs {Ethyl lactate (1) + Na_2Succinate [6] or Na_2Tartrate or NaKTartrate [6] or Na_3Citrate [6] or K_3Citrate [6] (2) + Water (3)}, at 298.15 K and 0.1 MPa.

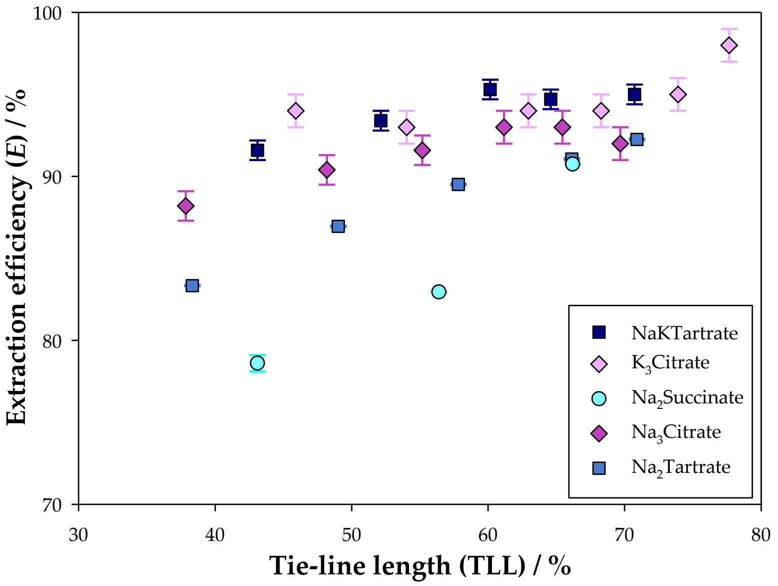

Figure S8. Extraction efficiencies (E) with tie-line length (TLL) for the extraction of resveratrol (RV) in the ATPSs {Ethyl lactate (1) + Na_2Succinate [6] or Na_2Tartrate or NaKTartrate [6] or Na_3Citrate [6] or K_3Citrate [6] (2) + Water (3)}, at 298.15 K and 0.1 MPa.

Table S1. Stage fraction and mean electrical charge (q) at different pH values for chlorogenic acid (CA), considering pK_a values of 3.50, 8.42 and 11.00 [7] [a].

pH	x_{CA^0}	$x_{CA^{-1}}$	$x_{CA^{-2}}$	$x_{CA^{-3}}$	q / e
0.00	1.00	0.00	0.00	0.00	0.00
0.25	1.00	0.00	0.00	0.00	0.00
0.50	1.00	0.00	0.00	0.00	0.00
0.75	1.00	0.00	0.00	0.00	0.00
1.00	1.00	0.00	0.00	0.00	0.00
1.25	0.99	0.01	0.00	0.00	-0.01
1.50	0.99	0.01	0.00	0.00	-0.01
1.75	0.98	0.02	0.00	0.00	-0.02
2.00	0.97	0.03	0.00	0.00	-0.03
2.25	0.95	0.05	0.00	0.00	-0.05
2.50	0.91	0.09	0.00	0.00	-0.09
2.75	0.85	0.15	0.00	0.00	-0.15
3.00	0.76	0.24	0.00	0.00	-0.24
3.25	0.64	0.36	0.00	0.00	-0.36
3.50	0.50	0.50	0.00	0.00	-0.50
3.75	0.36	0.64	0.00	0.00	-0.64
4.00	0.24	0.76	0.00	0.00	-0.76
4.25	0.15	0.85	0.00	0.00	-0.85
4.50	0.09	0.91	0.00	0.00	-0.91
4.75	0.05	0.95	0.00	0.00	-0.95
5.00	0.03	0.97	0.00	0.00	-0.97
5.25	0.02	0.98	0.00	0.00	-0.98
5.50	0.01	0.99	0.00	0.00	-0.99
5.75	0.01	0.99	0.00	0.00	-1.00
6.00	0.00	0.99	0.00	0.00	-1.00
6.25	0.00	0.99	0.01	0.00	-1.00
6.50	0.00	0.99	0.01	0.00	-1.01
6.75	0.00	0.98	0.02	0.00	-1.02
7.00	0.00	0.96	0.04	0.00	-1.04
7.25	0.00	0.94	0.06	0.00	-1.06
7.50	0.00	0.89	0.11	0.00	-1.11
7.75	0.00	0.82	0.18	0.00	-1.18
8.00	0.00	0.72	0.28	0.00	-1.28
8.25	0.00	0.60	0.40	0.00	-1.40
8.50	0.00	0.45	0.54	0.00	-1.55
8.75	0.00	0.32	0.68	0.00	-1.69
9.00	0.00	0.21	0.79	0.01	-1.80
9.25	0.00	0.13	0.86	0.02	-1.89
9.50	0.00	0.07	0.90	0.03	-1.95
9.75	0.00	0.04	0.91	0.05	-2.01
10.00	0.00	0.02	0.89	0.09	-2.07
10.25	0.00	0.01	0.84	0.15	-2.14
10.50	0.00	0.01	0.75	0.24	-2.23
10.75	0.00	0.00	0.64	0.36	-2.36
11.00	0.00	0.00	0.50	0.50	-2.50
11.25	0.00	0.00	0.36	0.64	-2.64
11.50	0.00	0.00	0.24	0.76	-2.76
11.75	0.00	0.00	0.15	0.85	-2.85
12.00	0.00	0.00	0.09	0.91	-2.91
12.25	0.00	0.00	0.05	0.95	-2.95
12.50	0.00	0.00	0.03	0.97	-2.97
12.75	0.00	0.00	0.02	0.98	-2.98
13.00	0.00	0.00	0.01	0.99	-2.99
13.25	0.00	0.00	0.01	0.99	-2.99
13.50	0.00	0.00	0.00	1.00	-3.00
13.75	0.00	0.00	0.00	1.00	-3.00
14.00	0.00	0.00	0.00	1.00	-3.00

[a] x_{CA^0}, $x_{CA^{-1}}$, $x_{CA^{-2}}$ and $x_{CA^{-3}}$ are the fractions of the species with electrical charges equal to 0, -1, -2 and -3 e, respectively. e stands for the elementary charge ($1.602 \cdot 10^{-19}$ C).

Table S2. Stage fraction and mean electrical charge (q) at different pH values for ferulic acid (FA), considering pK_a values of 4.50 and 8.92 [8] [a].

pH	x_{FA^0}	$x_{FA^{-1}}$	$x_{FA^{-2}}$	q / e
0.00	1.00	0.00	0.00	0.00
0.25	1.00	0.00	0.00	0.00
0.50	1.00	0.00	0.00	0.00
0.75	1.00	0.00	0.00	0.00
1.00	1.00	0.00	0.00	0.00
1.25	1.00	0.00	0.00	0.00
1.50	1.00	0.00	0.00	0.00
1.75	1.00	0.00	0.00	0.00
2.00	1.00	0.00	0.00	0.00
2.25	0.99	0.01	0.00	-0.01
2.50	0.99	0.01	0.00	-0.01
2.75	0.98	0.02	0.00	-0.02
3.00	0.97	0.03	0.00	-0.03
3.25	0.95	0.05	0.00	-0.05
3.50	0.91	0.09	0.00	-0.09
3.75	0.85	0.15	0.00	-0.15
4.00	0.76	0.24	0.00	-0.24
4.25	0.64	0.36	0.00	-0.36
4.50	0.50	0.50	0.00	-0.50
4.75	0.36	0.64	0.00	-0.64
5.00	0.24	0.76	0.00	-0.76
5.25	0.15	0.85	0.00	-0.85
5.50	0.09	0.91	0.00	-0.91
5.75	0.05	0.95	0.00	-0.95
6.00	0.03	0.97	0.00	-0.97
6.25	0.02	0.98	0.00	-0.98
6.50	0.01	0.99	0.00	-0.99
6.75	0.01	0.99	0.01	-1.00
7.00	0.00	0.99	0.01	-1.01
7.25	0.00	0.98	0.02	-1.02
7.50	0.00	0.96	0.04	-1.04
7.75	0.00	0.94	0.06	-1.06
8.00	0.00	0.89	0.11	-1.11
8.25	0.00	0.82	0.18	-1.18
8.50	0.00	0.72	0.28	-1.28
8.75	0.00	0.60	0.40	-1.40
9.00	0.00	0.45	0.55	-1.55
9.25	0.00	0.32	0.68	-1.68
9.50	0.00	0.21	0.79	-1.79
9.75	0.00	0.13	0.87	-1.87
10.00	0.00	0.08	0.92	-1.92
10.25	0.00	0.04	0.96	-1.96
10.50	0.00	0.03	0.97	-1.97
10.75	0.00	0.01	0.99	-1.99
11.00	0.00	0.01	0.99	-1.99
11.25	0.00	0.00	1.00	-2.00
11.50	0.00	0.00	1.00	-2.00
11.75	0.00	0.00	1.00	-2.00
12.00	0.00	0.00	1.00	-2.00
12.25	0.00	0.00	1.00	-2.00
12.50	0.00	0.00	1.00	-2.00
12.75	0.00	0.00	1.00	-2.00
13.00	0.00	0.00	1.00	-2.00
13.25	0.00	0.00	1.00	-2.00
13.50	0.00	0.00	1.00	-2.00
13.75	0.00	0.00	1.00	-2.00
14.00	0.00	0.00	1.00	-2.00

[a] x_{FA^0}, $x_{FA^{-1}}$ and $x_{FA^{-2}}$ are the fractions of the species with electrical charges equal to 0, -1 and -2 e, respectively. e stands for the elementary charge ($1.602 \cdot 10^{-19}$ C).

Table S3. Stage fraction and mean electrical charge (q) at different pH values for resveratrol (RV), considering pK_a values of 8.73, 9.56 and 10.88 [9] [a].

pH	x_{RV^0}	$x_{RV^{-1}}$	$x_{RV^{-2}}$	$x_{RV^{-3}}$	q / e
0.00	1.00	0.00	0.00	0.00	0.00
0.25	1.00	0.00	0.00	0.00	0.00
0.50	1.00	0.00	0.00	0.00	0.00
0.75	1.00	0.00	0.00	0.00	0.00
1.00	1.00	0.00	0.00	0.00	0.00
1.25	1.00	0.00	0.00	0.00	0.00
1.50	1.00	0.00	0.00	0.00	0.00
1.75	1.00	0.00	0.00	0.00	0.00
2.00	1.00	0.00	0.00	0.00	0.00
2.25	1.00	0.00	0.00	0.00	0.00
2.50	1.00	0.00	0.00	0.00	0.00
2.75	1.00	0.00	0.00	0.00	0.00
3.00	1.00	0.00	0.00	0.00	0.00
3.25	1.00	0.00	0.00	0.00	0.00
3.50	1.00	0.00	0.00	0.00	0.00
3.75	1.00	0.00	0.00	0.00	0.00
4.00	1.00	0.00	0.00	0.00	0.00
4.25	1.00	0.00	0.00	0.00	0.00
4.50	1.00	0.00	0.00	0.00	0.00
4.75	1.00	0.00	0.00	0.00	0.00
5.00	1.00	0.00	0.00	0.00	0.00
5.25	1.00	0.00	0.00	0.00	0.00
5.50	1.00	0.00	0.00	0.00	0.00
5.75	1.00	0.00	0.00	0.00	0.00
6.00	1.00	0.00	0.00	0.00	0.00
6.25	1.00	0.00	0.00	0.00	0.00
6.50	0.99	0.01	0.00	0.00	-0.01
6.75	0.99	0.01	0.00	0.00	-0.01
7.00	0.98	0.02	0.00	0.00	-0.02
7.25	0.97	0.03	0.00	0.00	-0.03
7.50	0.94	0.06	0.00	0.00	-0.06
7.75	0.90	0.09	0.00	0.00	-0.10
8.00	0.84	0.16	0.00	0.00	-0.16
8.25	0.74	0.25	0.01	0.00	-0.27
8.50	0.61	0.36	0.03	0.00	-0.42
8.75	0.45	0.47	0.07	0.00	-0.62
9.00	0.30	0.55	0.15	0.00	-0.86
9.25	0.17	0.55	0.27	0.01	-1.12
9.50	0.08	0.48	0.42	0.02	-1.37
9.75	0.03	0.36	0.56	0.04	-1.61
10.00	0.01	0.24	0.66	0.09	-1.82
10.25	0.00	0.14	0.69	0.16	-2.01
10.50	0.00	0.07	0.65	0.27	-2.19
10.75	0.00	0.04	0.55	0.41	-2.37
11.00	0.00	0.02	0.42	0.56	-2.54
11.25	0.00	0.01	0.30	0.70	-2.69
11.50	0.00	0.00	0.19	0.80	-2.80
11.75	0.00	0.00	0.12	0.88	-2.88
12.00	0.00	0.00	0.07	0.93	-2.93
12.25	0.00	0.00	0.04	0.96	-2.96
12.50	0.00	0.00	0.02	0.98	-2.98
12.75	0.00	0.00	0.01	0.99	-2.99
13.00	0.00	0.00	0.01	0.99	-2.99
13.25	0.00	0.00	0.00	1.00	-3.00
13.50	0.00	0.00	0.00	1.00	-3.00
13.75	0.00	0.00	0.00	1.00	-3.00
14.00	0.00	0.00	0.00	1.00	-3.00

[a] x_{RV^0}, $x_{RV^{-1}}$, $x_{RV^{-2}}$ and $x_{RV^{-3}}$ are the fractions of the species with electrical charges equal to 0, -1, -2 and -3 e, respectively. e stands for the elementary charge ($1.602 \cdot 10^{-19}$ C).

References

[1] Kamalanathan, I.; Petrovski, Z.; Branco, L. C.; Najdanovic-Visak, V. Novel aqueous biphasic system based on ethyl lactate for sustainable separations: Phase splitting mechanism. *J. Mol. Liq.* **2018**, *262*, 37-45. www.doi.org/10.1016/j.molliq.2018.03.119

[2] Requejo, P. F.; Velho, P.; Gómez, E.; Macedo, E. A. Study of liquid–liquid equilibrium of aqueous two-phase systems based on ethyl lactate and partitioning of rutin and quercetin. *Ind. Eng. Chem. Res.* **2020**, *59*, 21196-21204. www.doi.org/10.1021/acs.iecr.0c02664

[3] Velho, P.; Perestrelo, G.; Macedo, E. A. Partition of Chlorogenic and Nicotinic Acids Using Eco-Friendly ATPSs Containing Ethyl Lactate at 298.15 K and 0.1 MPa. *J. Chem. Eng. Data* **2024**, *69*, 3075-3084. www.doi.org/10.1021/acs.jced.4c00197

[4] Rebelo, C. S.; Velho, P.; Macedo, E. A. eNRTL modelling and partition of phenolics in the ATPSs {ethyl lactate (1) + potassium sodium tartrate or disodium succinate (2) + water (3)} at 298.2 K and 0.1 MPa. *Fluid Phase Equilib.* **2024**, *582*, 114087. www.doi.org/10.1016/j.fluid.2024.114087

[5] Velho, P.; Rebelo, C. S.; Macedo, E. A. Extraction of Gallic Acid and Ferulic Acid for Application in Hair Supplements. *Molecules* **2023**, *28*, 2369. www.doi.org/10.3390/molecules28052369

[6] Rebelo, C. S.; Velho, P.; Macedo, E. A. Partition Studies of Resveratrol in Low-Impact ATPS for Food Supplementation. *Ind. Eng. Chem. Res.* **2024**, *63*, 2885-2894. www.doi.org/10.1021/acs.iecr.3c03969

[7] Maegawa, Y.; Sugino, K.; Sakurai, H. Identification of free radical species derived from caffeic acid and related polyphenols. *Free Radical Res.* **2007**, *41*, 110-119. www.doi.org/10.1080/10715760600943892

[8] Borges, F.; Lima, J. L. F. C.; Pinto, I.; Reis, S.; Siquet, C. Application of a Potentiometric System with Data-Analysis Computer Programs to the Quantification of Metal-Chelating Activity of Two Natural Antioxidants: Caffeic Acid and Ferulic Acid. *Helv. Chim. Acta* **2003**, *86*, 3081-3087. www.doi.org/10.1002/hlca.200390250

[9] Brittes, J.; Lúcio, M.; Nunes, C.; Lima, J. L. F. C.; Reis, S. Effects of resveratrol on membrane biophysical properties: relevance for its pharmacological effects. *Chem. Phys. Lipids* **2010**, *163*, 747-754. www.doi.org/10.1016/j.chemphyslip.2010.07.004
